# Machine Learning Applied to Omics Datasets Predicts Mortality in Patients with Alcoholic Hepatitis

**DOI:** 10.3390/metabo12010041

**Published:** 2022-01-05

**Authors:** Bei Gao, Tsung-Chin Wu, Sonja Lang, Lu Jiang, Yi Duan, Derrick E. Fouts, Xinlian Zhang, Xin-Ming Tu, Bernd Schnabl

**Affiliations:** 1 School of Marine Sciences, Nanjing University of Information Science and Technology, Nanjing 210044, China; wintergb@hotmail.com; 2 Department of Medicine, University of California San Diego, La Jolla, CA 92093, USA; slang@health.ucsd.edu (S.L.); jianglu@xinhuamed.com.cn (L.J.); yid003@health.ucsd.edu (Y.D.); 3 Department of Mathematics, University of California San Diego, San Diego, CA 92093, USA; tswu@health.ucsd.edu; 4 Division of Biostatistics and Bioinformatics, Herbert Wertheim School of Public Health, University of California San Diego, San Diego, CA 92093, USA; xizhang@health.ucsd.edu (X.Z.); x2tu@health.ucsd.edu (X.-M.T.); 5 Department of Medicine, VA San Diego Healthcare System, San Diego, CA 92161, USA; 6 J. Craig Venter Institute, Rockville, MD 20850, USA; dfouts@jcvi.org

**Keywords:** machine learning, mycobiome, virome, microbiota, metabolomics

## Abstract

Alcoholic hepatitis is a major health care burden in the United States due to significant morbidity and mortality. Early identification of patients with alcoholic hepatitis at greatest risk of death is extremely important for proper treatments and interventions to be instituted. In this study, we used gradient boosting, random forest, support vector machine and logistic regression analysis of laboratory parameters, fecal bacterial microbiota, fecal mycobiota, fecal virome, serum metabolome and serum lipidome to predict mortality in patients with alcoholic hepatitis. Gradient boosting achieved the highest AUC of 0.87 for both 30-day mortality prediction using the bacteria and metabolic pathways dataset and 90-day mortality prediction using the fungi dataset, which showed better performance than the currently used model for end-stage liver disease (MELD) score.

## 1. Introduction

Alcohol use disorder is a major healthcare burden. Common consequences of heavy alcohol consumption include a wide spectrum of liver diseases, such as alcohol-associated steatosis, fibrosis and cirrhosis [1]. Alcoholic hepatitis represents the most severe manifestation of alcohol-related liver disease, with an annual incidence rate of 34 per million in women and 46 per million in men [2]. As a life-threatening disease, alcoholic hepatitis is associated with a mortality rate of 15%, 24%, and 56% for 28-day, 84-day and 5-year mortality, respectively [2]. Severe alcoholic hepatitis is associated with a very high 90-day mortality of up to 75% [3]. Therefore, it is crucial to accurately determine the prognosis of patients presenting with acute alcoholic hepatitis. Early identification of alcoholic hepatitis patients at greatest risk of death is extremely important for the stratification of patients towards proper treatments, such as corticosteroids, liver transplantation or clinical trials.

Alcohol-associated liver disease is transmissible via fecal microbiota transfer in mice [4], and a small clinical trial showed survival benefits in patients with severe alcoholic hepatitis receiving daily fecal microbiota transplantation for 7 days from a healthy donor [5]. Thus, the intestinal microbiota is very important for development and disease outcome in patients with alcoholic hepatitis. The bacterial microbiota, fungal mycobiota and virome are involved in pathogenesis of alcoholic hepatitis [6,7,8,9]. Alcoholic hepatitis is also accompanied by a profound dysfunction of the intestinal barrier leading to bacterial translocation to the liver and worse disease outcome [10]. Common serum biomarkers used to evaluate gut barrier dysfunction include anti-Saccharomyces cerevisiae antibodies (ASCA), zonulin and lipopolysaccharide binding protein (LBP). ASCA are systemic antibodies against fungal antigens [11]. Serum zonulin is a surrogate marker for intestinal permeability [12,13]. LBP is synthesized in response to translocated LPS and serves as an additional biomarker for gut barrier dysfunction [14].

As a subset of artificial intelligence, machine learning is an umbrella term for a variety of important computational tools for early diagnosis and prognosis, which includes different classification models, such as gradient boosting, random forest, and support vector machine. Machine learning generates predictive models effectively through the detection of hidden patterns within big datasets. Given that a lot of variables could affect the clinical outcome, it is often difficult for a physician to predict a given outcome to ascertain. Machine learning algorithms could better incorporate various risk factors to identify nuanced interactions between outcomes and variables, which allows them to find new patterns between risk factors. Predicting clinical outcomes using a profiling dataset with a large number of variables has drawn great interest over the past years. For instance, clinical data and microbiota based multi-omics have been used to predict outcome or severity of diseases such as nonalcoholic fatty liver disease (NAFLD) and nonalcoholic steatohepatitis (NASH) [15,16].

In the present study, we demonstrate the use of machine learning tools to predict 30-day and 90-day mortality in patients with alcoholic hepatitis using clinical data. In particular, we compare four popular models: gradient boosting, random forest, support vector machine, and logistic regression models. Our second aim is to identify key features associated with high mortality from multi-omics with a particular focus on datasets derived from a global characterization of the gut microbiota.

## 2. Results

### 2.1. Mortality Prediction with Clinical Data in Patients with Alcoholic Hepatitis

**Figure 1:**
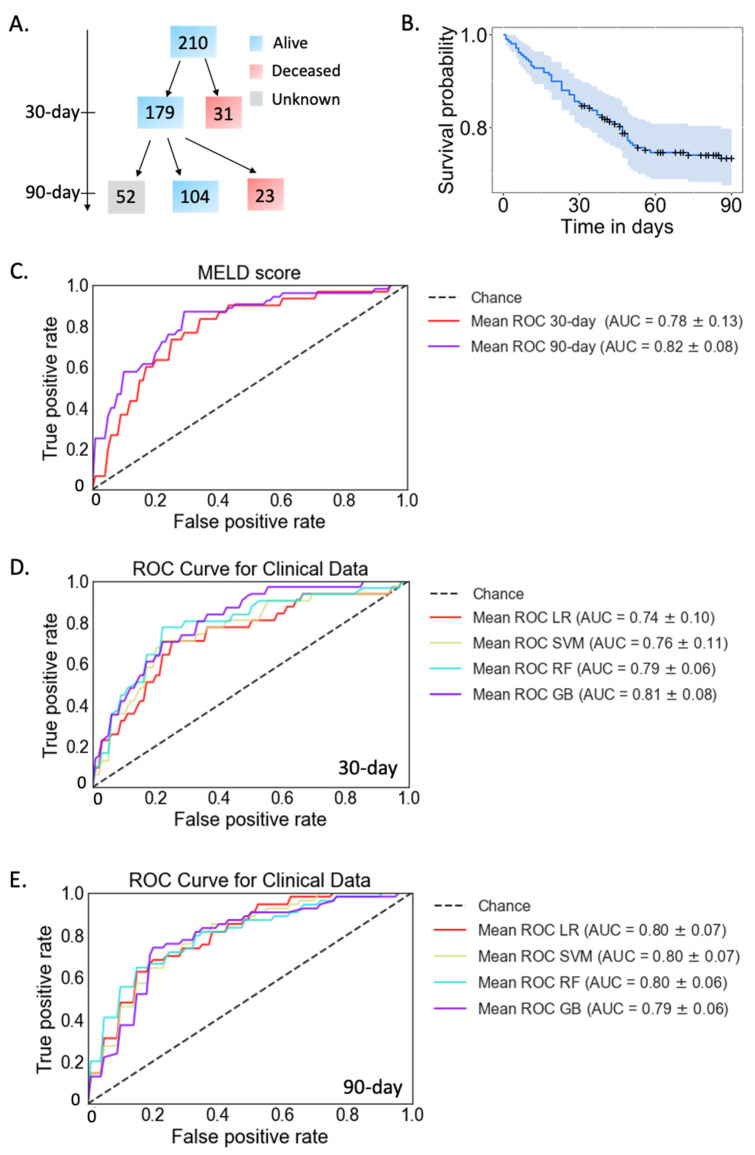
Mortality prediction with clinical parameters in patients with alcoholic hepatitis. (**A**) A total of 210 patients were included in this study. 31 died within 30-day and 179 patients were alive, 23 patients died within 90 days. A total of 104 patients survived at 90 days. The remaining 52 patients were lost to follow-up. Blue: alive. Red: deceased. Grey: unknown status. (**B**) Survival probability within 90 days. Confidence intervals are shown in light blue. +: Patients lost to follow-up were censored at the time they were last seen alive. (**C**) Prediction of 30-day (red line, AUC = 0.78) and 90-day mortality using MELD score (purple line, AUC = 0.82). (**D**) Four models for 30-day mortality prediction in patients with alcoholic hepatitis using clinical data. Day-30 deceased group *n* = 31, alive group *n* = 179. (**E**) Four models for 90-day mortality prediction in patients with alcoholic hepatitis using clinical data. Day-90 deceased group *n* = 54, alive group *n* = 104. GB: gradient boosting, LR: logistic regression, RF: random forest, SVM: support vector machine.

A total of 210 patients with alcoholic hepatitis were included in this study (Table 1). Of these, 31 (14.8%) patients died within 30 days. Among 179 patients alive at day 30, 23 patients (12.8%) died within 90 days, 104 patients were alive at 90 days, while the remaining 52 patients were lost to follow-up (Figure 1A,B). The model of end-stage liver disease (MELD) score is currently used in clinical practice to predict mortality in alcoholic hepatitis patients. In our dataset, the area under the receiver operating characteristic curve (AUC) was 0.78 and 0.82 for the logistic regression model when predicting 30-day and 90-day mortality, respectively, using MELD score (Figure 1C).

To assist physicians to make clinical decisions more precisely, we developed four models using 11 routine clinical laboratory variables to predict mortality in patients with alcoholic hepatitis (Table 2). From now on, we refer to these 11 clinical laboratory variables as Clinical data. These 11 variables were selected based on the availability of clinical data collected and the missing-value rate. Only clinical parameters with a missing-value rate less than 20% were selected. When predicting 30-day mortality, the AUC achieved 0.74–0.81 using four different models: gradient boosting, logistic regression, random forest or support vector machine (Figure 1D). The AUC for 90-day mortality prediction achieved 0.79–0.80 using these models (Figure 1E). Among these four models, gradient boosting attained the highest AUC for 30-day mortality prediction, and the other three models attained the highest AUC for 90-day mortality prediction. In particular, the prediction of 30-day mortality in patients with alcoholic hepatitis from the random forest and gradient boosting was slightly better than the currently used MELD score in clinical practice (Table 3).

### 2.2. Selected Variables from Multi-Omics Datasets

**Figure 2:**
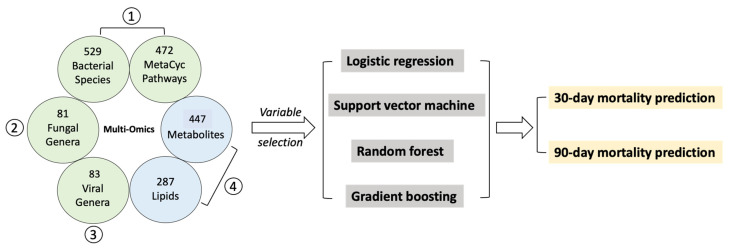
Multi-omics datasets used for mortality prediction in patients with alcoholic hepatitis. A total of (1) 529 fecal bacterial species and 472 MetaCyc pathways, (2) 81 fecal fungal genera, (3) 83 fecal viral genera, (4) 447 serum metabolites and 287 serum lipids were included.

To further improve the performance of mortality prediction for patients with alcoholic hepatitis, we collected multi-omics data, including fecal bacterial microbiome, fecal fungal mycobiome, fecal virome, serum metabolome and lipidome (Figure 2). Due to limited sample availability, multi-omics data were collected from only a subset of the patient cohort, and multiple imputation was applied to the multi-omics data to preserve all samples having missing values. After multiple imputation, we used random forest to select variables with the top 11 average feature importance in each multi-omics data, so the number of variables included in the models using the multi-omics data and using the clinical data was the same. The 11 selected variables are listed in Table 2. When implementing the random forest, we forced the component variables used to calculate the MELD score (creatinine, bilirubin, international normalized ratio, and sodium) to be selected for splitting at each node in the trees. Then, using these 11 selected features for each multi-omics dataset, we built gradient boosting, logistic regression, random forest or support vector machine models to predict short-term mortality. In order to compare the performance of each model, we calculated the AUC score for the logistic regression model using MELD score only based on the same subset of patients for each multi-omics dataset.

### 2.3. Fecal Bacteria and MetaCyc Pathways

**Figure 3:**
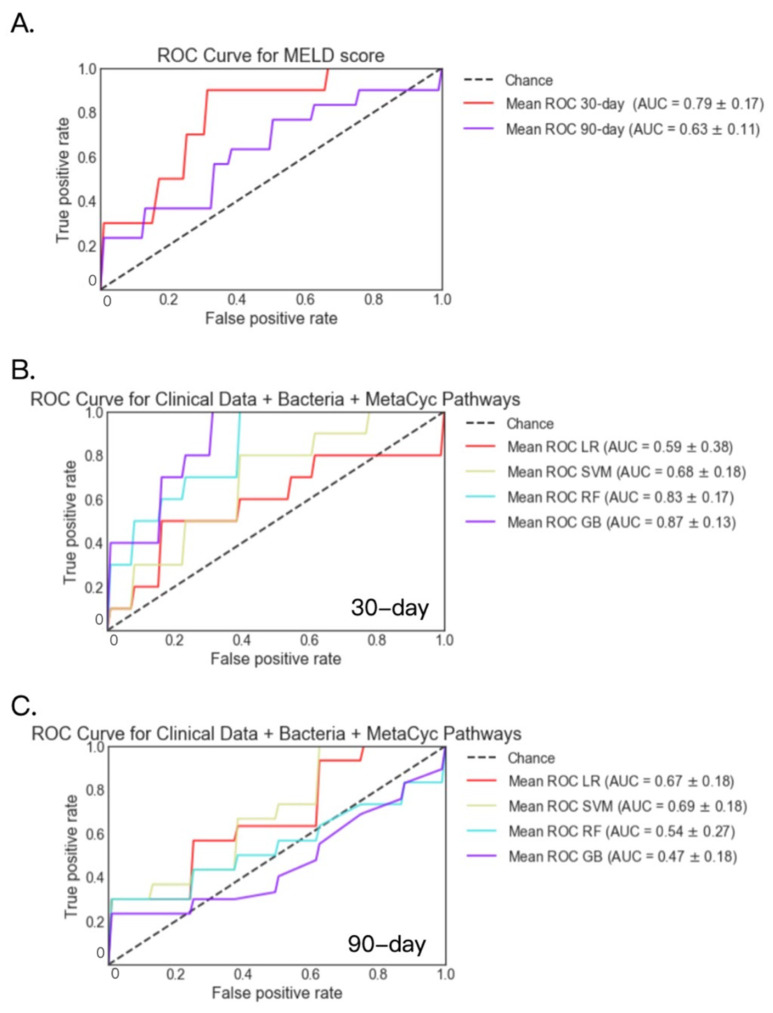
30- and 90-day mortality prediction using fecal bacteria, MetaCyc pathways and clinical data in patients with alcoholic hepatitis. (**A**) 30- and 90-day mortality prediction using MELD score. (**B**) 30-day mortality prediction using fecal bacteria, Metacyc pathways and clinical data. Deceased group *n* = 8; alive group *n* = 65. (**C**) 90-day mortality prediction using fecal bacteria, Metacyc pathways and clinical data. Deceased group *n* = 13; alive group *n* = 40. GB: gradient boosting, LR: logistic regression, RF: random forest, SVM: support vector machine.

Bacteria and Metacyc pathways were available for 73 patients at 30 days and for 53 patients at 90 days. For these patients, AUCs were 0.79 and 0.63 when predicting 30-day and 90-day mortality using the logistic regression model with MELD score only, respectively (Figure 3A). Among the four models, bacteria, metabolic (MetaCyc) pathways and clinical data achieved the highest AUC of 0.87 for 30-day mortality using the gradient boosting model, and 0.69 for 90-day mortality using the support vector machine model (Figure 3B,C), both of which were higher than AUC based on MELD score only (Table 3).

### 2.4. Fecal Fungal Datasets

**Figure 4:**
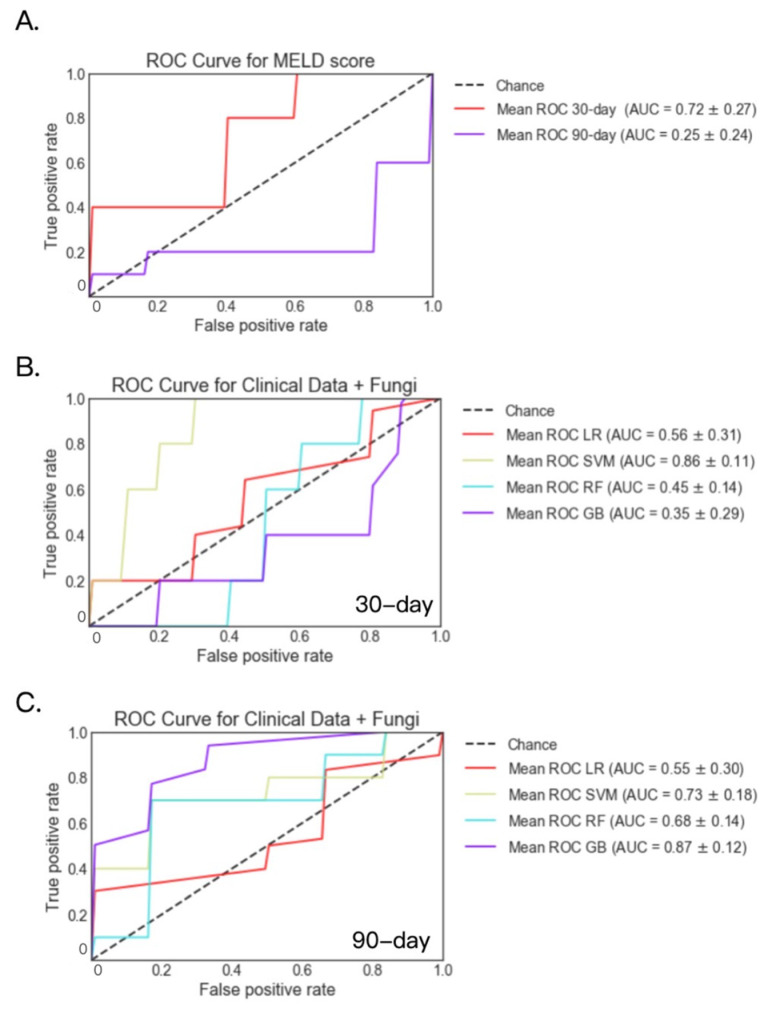
30- and 90-day mortality prediction using fecal fungi and clinical data in patients with alcoholic hepatitis. (**A**) 30- and 90-day mortality prediction using MELD score. (**B**) 30-day mortality prediction using fecal fungi and clinical data. Deceased group *n* = 5; alive group *n* = 49. (**C**) 90-day mortality prediction using fecal fungi and clinical data. Deceased group *n* = 9; alive group *n* = 30. GB: gradient boosting, LR: logistic regression, RF: random forest, SVM: support vector machine.

Fecal fungi were available for 54 patients at 30-day and 39 patients at 90-day. For these patients, AUC was 0.72 and 0.25 for the logistic regression model when predicting 30-day and 90-day mortality using MELD score only, respectively (Figure 4A). Applying the support vector machine model to fecal fungi and clinical data, the highest AUC of 0.86 was achieved for 30-day mortality. Meanwhile, the highest AUC of 0.87 was achieved for 90-day mortality by the gradient boosting model (Figure 4B,C). All four models based on fecal fungi and clinical laboratory data performed better when predicting 90-day mortality than the logistic regression model based on MELD score (Table 3).

### 2.5. Fecal Viral Datasets

**Figure 5:**
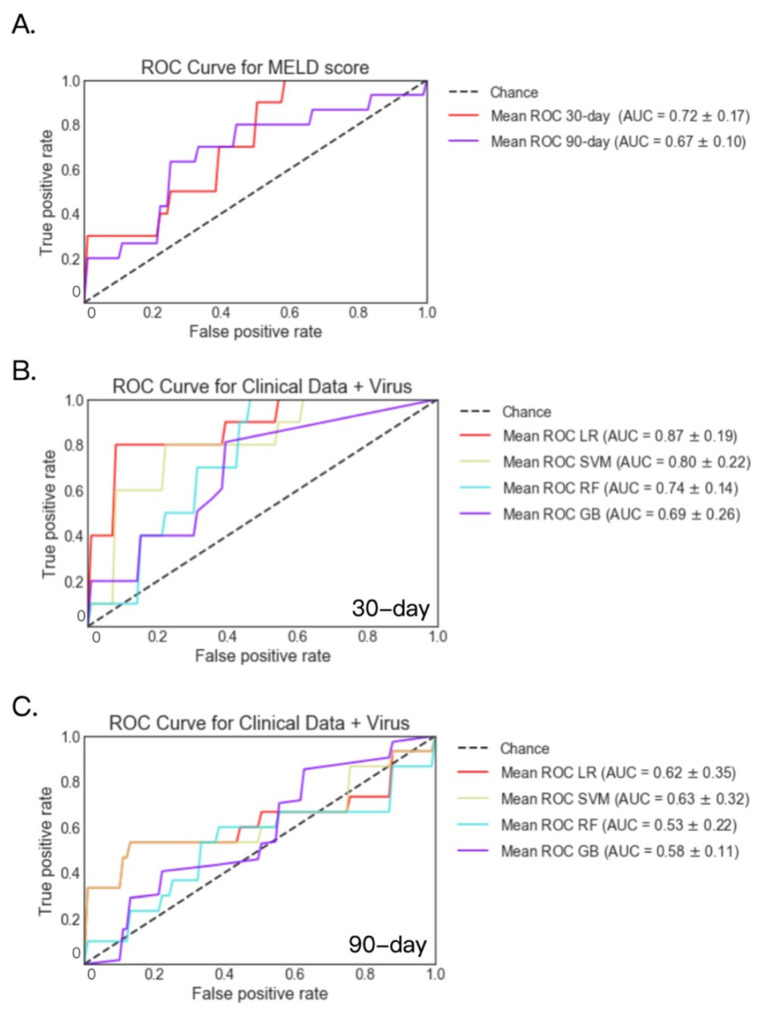
30- and 90-day mortality prediction using fecal viruses and clinical data in patients with alcoholic hepatitis. (**A**) 30- and 90-day mortality prediction using MELD score. (**B**) 30-day mortality prediction using fecal viruses and clinical data. Deceased group *n* = 8; alive group *n* = 68. (**C**) 90-day mortality prediction using fecal viruses and clinical data. Deceased group *n* = 14; alive group *n* = 42. GB: gradient boosting, LR: logistic regression, RF: random forest, SVM: support vector machine.

Fecal virome analysis was available for 76 patients at 30-day and 56 patients at 90-day. For these patients, AUC was 0.72 and 0.67 for the logistic regression model when predicting 30-day and 90-day mortality using MELD score only, respectively (Figure 5A). Among the four models, viral and clinical laboratory data achieved the highest AUC of 0.87 for 30-day mortality using the logistic regression model, and achieved the highest AUC of 0.63 for 90-day mortality using the support vector machine model (Figure 5B,C). The logistic regression, support vector machine, and random forest models based on the viral and clinical laboratory data performed better when predicting 30-day mortality than the logistic regression model based on MELD score (Table 3).

### 2.6. Serum Metabolites and Lipids

**Figure 6:**
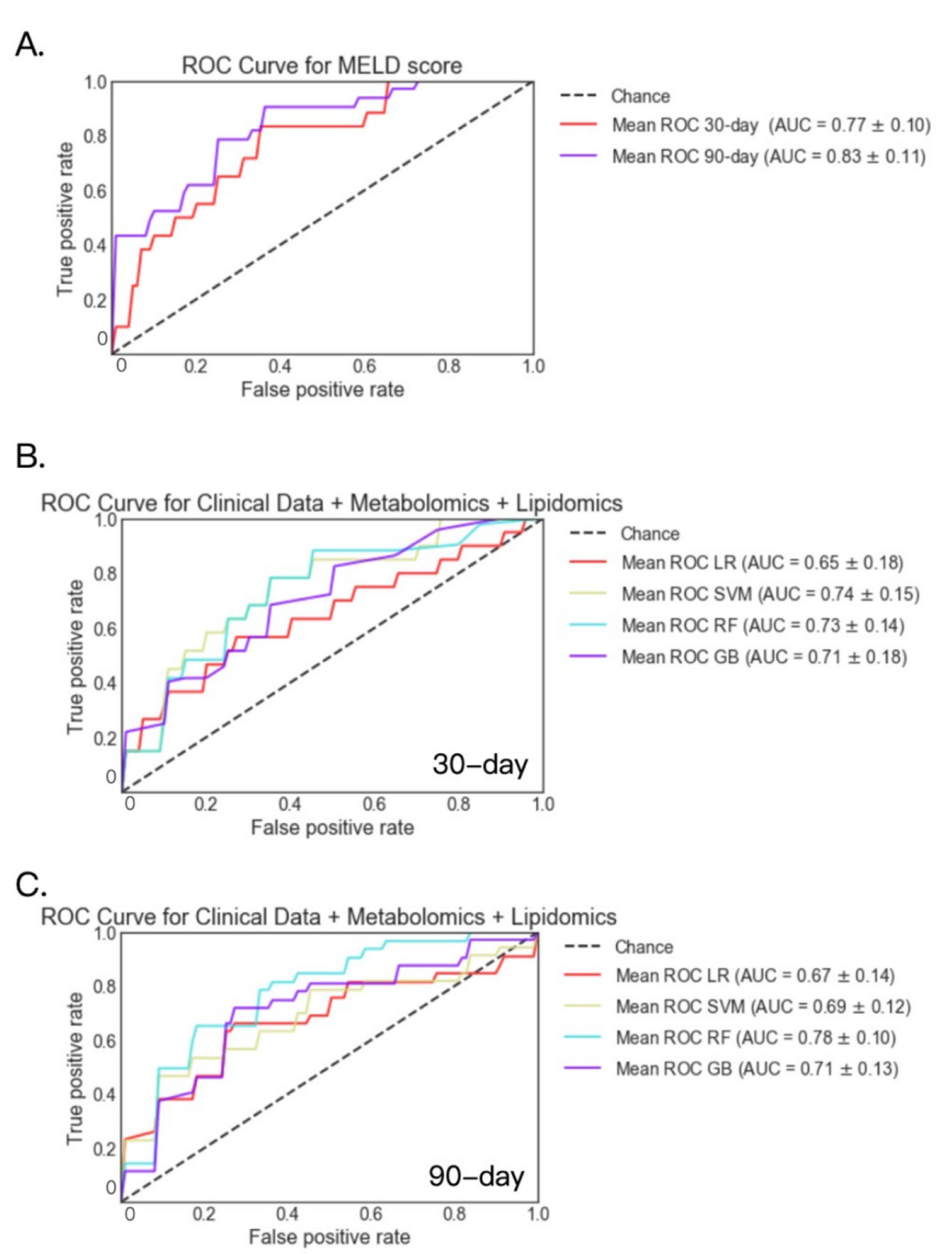
30- and 90-day mortality prediction using serum metabolites, serum lipids and clinical data in patients with alcoholic hepatitis. (**A**) 30- and 90-day mortality prediction using MELD score. (**B**) 30-day mortality prediction using serum metabolites, serum lipids and clinical data. Deceased group *n* = 19; alive group *n* = 99. (**C**) 90-day mortality prediction using serum metabolites, serum lipids and clinical data. Deceased group *n* = 33; alive group *n* = 57. GB: gradient boosting, LR: logistic regression, RF: random forest, SVM: support vector machine.

Serum metabolites and lipids were available for 118 patients at 30 days and 90 patients at 90 days. For these patients, AUC was 0.77 and 0.83 for the logistic regression model when predicting 30-day and 90-day mortality using the MELD score only, respectively (Figure 6A). Among the four models, metabolites and lipids achieved the highest AUC of 0.74 for 30-day mortality using the support vector machine model, and achieved the highest AUC of 0.78 for 90-day mortality using the random forest model (Figure 6B,C).

### 2.7. ASCA, Zonulin and LBP

**Figure 7:**
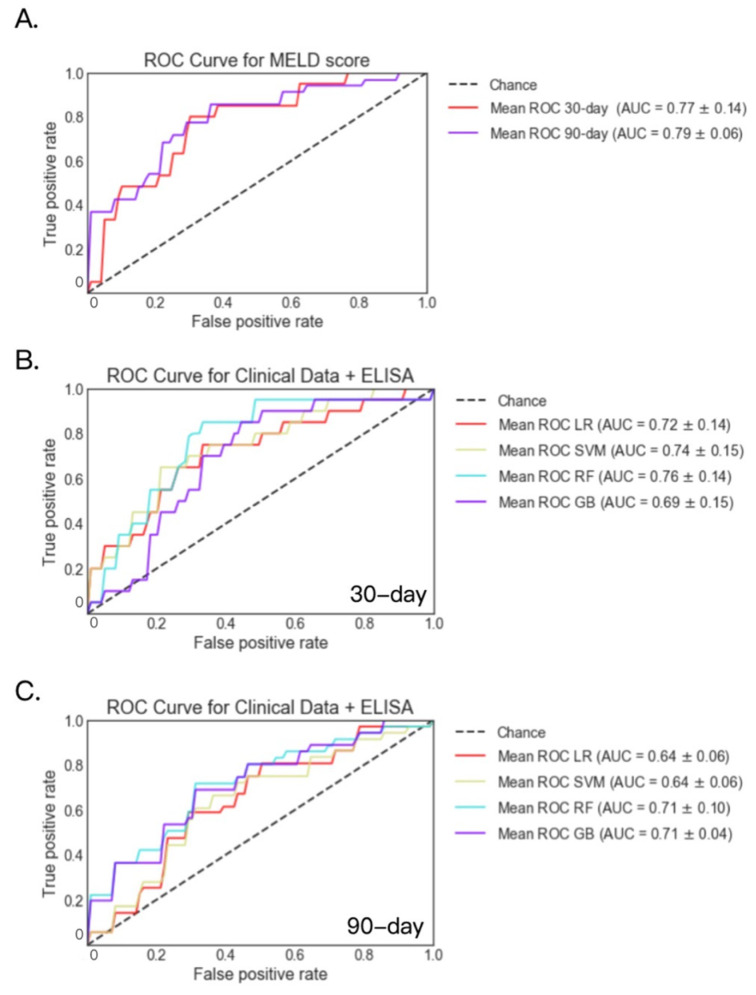
30- and 90-day mortality prediction using serum biomarkers and clinical data in patients with alcoholic hepatitis. (**A**) 30- and 90-day mortality prediction using MELD score. (**B**) 30-day mortality prediction using serum biomarkers and clinical data. Decreased group *n* = 20; alive group *n* = 118. (**C**) 90-day mortality prediction using serum biomarkers and clinical data. Decreased group *n* = 36; alive group *n* = 68. GB: gradient boosting, LR: logistic regression, RF: random forest, SVM: support vector machine.

In addition to multi-omics datasets, we also evaluated routine laboratory parameters together with serum biomarkers of gut barrier dysfunction, ASCA, zonulin and LBP. These data were available for 138 patients at 30 days and 114 patients at 90 days. For these patients, AUC was 0.77 and 0.79 for the logistic regression model when predicting 30-day and 90-day mortality using the MELD score only, respectively (Figure 7A). Among the four models, the highest AUC was 0.76 for 30-day mortality using the random forest model, and 0.71 for 90-day mortality using the random forest and gradient boosting models (Figure 7B,C).

A summary of AUC scores for each dataset is shown in Table 3. Multi-omics or serum biomarkers combined with routine clinical laboratory parameters improved the performance for the prediction of 30- and 90-day mortality in patients with alcoholic hepatitis, with the highest AUC achieved being 0.87 (gradient boosting using bacteria, Metacyc pathways and clinical data, as well as logistic regression using viral and clinical data) and 0.87 (gradient boosting model using fungi and clinical data), respectively.

## 3. Discussion

The identification of patients with alcoholic hepatitis at greatest risk of death is necessary for treatment stratification towards early liver transplantation, prednisolone therapy, clinical trial or supportive care. Invasive testing with liver biopsy can lead to increased morbidity, and is currently recommended to confirm the diagnosis of alcoholic hepatitis only in the presence of potential confounding factors or if treatment with immunosuppressive therapy is considered [17]. Therefore, non-invasive scoring systems are important, and various prognostic clinical models have been developed and applied to patients to assess the severity of alcoholic hepatitis. The AUCs for the discriminant function (DF) were significantly lower than for MELD, the age, serum bilirubin, international normalized ratio and serum creatinine (ABIC) score, and Glasgow alcoholic hepatitis score (GAHS) for both 28- and 90-day outcomes: 90-day values were 0.670, 0.704, 0.726 and 0.713, respectively [18].

The implementation of machine learning models has rapidly increased in the biomedical field including liver diseases in recent years [15,16,19,20]. To predict cirrhosis in patients with non-alcoholic fatty liver disease (NAFLD), AUC achieved 0.91 when using random forest machine learning algorithm to integrate shotgun metagenomic and untargeted metabolomic profiles [21]. However, a promising model for mortality prediction has not been applied to patients with alcoholic hepatitis. In the present study, we developed four models to predict short-term mortality, and some of them showed better performance than the currently used MELD score. Especially, the gradient boosting analysis of bacteria and metabolic pathways datasets achieved the highest AUC of 0.87 for 30-day mortality prediction. Among the selected bacteria and metabolic pathways used for the 30-day mortality prediction, 6 pathways were related to purine nucleoside biosynthesis. Nucleotide biosynthesis has been reported to be critical for the growth of bacteria in human blood [22]. The causal relationship between microbial nucleoside biosynthesis and mortality requires further investigation.

In addition to multi-omics study design and comparison of four models, the multi-center study design is another strength of this study, which recruited patients from diverse geographical origins. One limitation of this study was the relatively small sample size, given the complexity of the machine learning pipelines used. Despite using a test and validation cohort in our study, external validation with a larger number of patients is required to confirm our prediction model. Another limitation is that omics are not yet readily usable in routine clinical practice unless these methods become less expensive and more standardized.

In summary, this is the first comprehensive study to predict short-term mortality using different machine learning algorithms with multi-omics data covering not only serum metabolites, serum lipids and fecal bacteria, but also fecal fungi and viruses, which were not well studied in alcoholic hepatitis. This model is helpful for physicians to identify patients with greatest risk and make better clinical decisions for patients with alcoholic hepatitis.

## 4. Materials and Methods

### 4.1. Patients

A total of 210 patients diagnosed with alcoholic hepatitis were recruited from 10 institutions in the United States, Canada and Europe. The clinical picture was consistent with alcoholic hepatitis in all patients. The patient cohort has been described previously [6,8,23]. The inclusion criteria for alcoholic hepatitis were: 1. active alcohol use (>50 g/day for men and >40 g/day for women) in the last 3 months; 2. aspartate aminotransferase (AST) >alanine aminotransferase (ALT) and total bilirubin >3 mg/dL in the past 3 months; 3. liver biopsy and/or clinical picture consistent with alcoholic hepatitis. The exclusion criteria were: 1. autoimmune liver disease (ANA > 1/320); 2. chronic viral hepatitis; 3. hepatocellular carcinoma; 4. complete portal vein thrombosis; 5. extrahepatic terminal disease; 6. pregnancy; 7. lack of signed informed consent. Liver biopsies were performed only if indicated as part of routine clinical care for the purpose of alcoholic hepatitis diagnosis. For patients who underwent liver biopsy, the liver histology was in line with the diagnosis of alcoholic hepatitis. The protocol was approved by the Ethics Committee of each participating center. Written informed consent was obtained from each subject. The MELD score was calculated for all alcoholic hepatitis patients whose required variables were available. Eleven clinical parameters were evaluated in the random forest model to predict the 30-day and 90-day mortality, including age, creatinine, bilirubin, albumin, international normalized ratio, alanine transaminase, alkaline phosphatase, platelet count, white blood cell count, aspartate transaminase, and sodium.

### 4.2. Shotgun Metagenomics

DNA was extracted from stool samples collected from 73 patients. DNA extraction and library preparation were performed as described previously [24]. Shot-gun metagenomics sequencing was performed on an Illumina HiSeq 4000, generating 150bp paired-end reads. KneadData version 0.7.2 was used for quality control. Metagenomic Phylogenetic Analysis 2 (MetaPhlAn2) version 2.7.7 was used for the profiling of the composition of the microbial community [25]. The HMP Unified Metabolic Analysis Network 2 (HUMAnN2) version 0.11.1 was used for the profiling of microbial pathways [26]. The MetaCyc database was used for microbial pathway analysis [27]. Each of the HUMAnN2 abundance outputs was normalized into relative abundance (the counts for each sample sum to 100).

### 4.3. Mycobiome Analysis

Fecal mycobiomes were evaluated using internal transcribed spacer (ITS) sequencing targeting fungal ITS1 region from 54 patients. Fungal ITS sequencing was performed using Illumina MiSeq V2 kit, 300 cycles using primers. Primers, PCR conditions and data processing were described in our previous study [8].

### 4.4. Viral Metagenomics

Virus-like particles were isolated from fecal samples collected from 76 patients using differential filtration techniques followed by metagenomic sequencing. Viromes were prepared using the NetoVIR protocol, with minor modifications [28]. Briefly, resuspended fecal samples were filtered using a 0.8 μm (PES) filter (Sartorius). The remaining supernatant was subjected to lysis followed by viral DNA and RNA extraction. Library preparation was performed as described previously [9]. Clumpify and Kneaddata were used for the quality control of raw sequence reads. The PathSeq pipeline was used for the read alignment and taxonomy assignment [29].

### 4.5. Untargeted Metabolomics and Lipidomics

Serum metabolome and lipidome from 132 patients were analyzed by multi-platforms, including gas chromatography-time of flight mass spectrometry (GC-TOF MS), hydrophilic interaction liquid chromatography (HILIC) with quadrupole orbital ion trap high field mass spectrometry (Q-Exactive HF MS), and CSH-Q-Exactive HF MS. Sample extraction, data acquisition and data processing were performed as described in our previous study [30]. Briefly, ChromaTOF version 4.50 was used for baseline subtraction, deconvolution and peak detection for GC-MS raw data. Binbase version 5.0.3 was used for metabolite annotation and reporting [31]. For LC-MS raw data, MS-DIAL was used for peak picking, alignment, deconvolution and identification [32]. The level of confidence in the identification was level 3 [33]. MS-FLO was used for the identification of ion adducts, duplicate peaks and isotopic features [34]. For both the HILIC and lipidomics datasets, retention time-m/z libraries and MS/MS spectra databases were used for compound identification, which were uploaded to MassBank of North America.

### 4.6. Enzyme Linked Immunosorbent Assay (ELISA)

Anti-saccharomyces cerevisiae antibody (ASCA)-IgG, zonulin and lipopolysaccharide binding protein (LBP) levels were measured in the serum samples collected from 132 patients using different ELISA kits, as described previously [8].

### 4.7. Machine Learning Models

The predictive power with eleven clinical parameters, multi-omics datasets, and three markers for intestinal permeability was evaluated for the short-term mortality prediction in patients with alcoholic hepatitis. Logistic regression (LR) [35], support vector machine (SVM) [36], random forest (RF) [37], and gradient boosting (GB) [38] were built using functions from scikit-learn in Python. Before any data preprocessing, we divided the datasets into 5 folds using stratified 5-fold cross-validation (CV). Multivariate imputation by chained equations (MICE) was used to impute the missing values in each feature [39]. To avoid data leakage from the training set to the test set, MICE was only applied on the training set inside each CV iteration, and the test set was imputed by the fitted model of MICE on the training set. In order to deal with the class imbalance and promote the performance of the models, after imputation, the synthetic minority oversampling technique (SMOTE) was used to oversample the minor class in the training set only to obtain balanced data [40]. When the multi-omics datasets were used for the short-term mortality prediction, random forest from the ranger Package in R was additionally applied to the training set before performing SMOTE to select 11 variables based on the average feature importance over 5 CV iterations mentioned above [41]. The component variables used to calculate the MELD score were always selected for splitting at each node in trees when building a ranger random forest. For the main models (LR, SVM, RF, or GB), the default setting was used when building LR and SVM, and the number of trees and the maximum tree depth were chosen for tuning when building RF and GB. In order to choose the best set of hyperparameters in RF and GB, grid search with stratified 4-fold CV (inner CV) was performed in each CV iteration using the original training set (the one before doing MICE). In RF, the number of trees chosen was from 100, 200, 300, 400, and the maximum tree depth chosen was from 5, 10, 15, 20. In GB, the number of trees was chosen from 100, 200, 300, 400, and the maximum tree depth chosen was from 1, 2, 3, 4. In each inner CV iteration, MICE, feature selection, and SMOTE were performed again on the training subset to avoid data leakage from the training subset to the validation set, and the imputation model from this second MICE was used to impute the validation set. A set of hyperparameters with the highest F1 score evaluated on the validation set was chosen (40), then we fit the main model with this best set of hyperparameters using the training set (the one after doing the SMOTE in Supplementary Figure S1A) and assessed the model on the test set for each outer CV iteration. The final model performance was the average of model performance for each outer CV iteration. ROC was used to evaluate performances for the short-term mortality prediction in patients with alcoholic hepatitis. A more comprehensive description on the details of the procedures is shown in Supplementary Figure S1.

## Figures and Tables

**Table 1:** Characteristics of patients with alcoholic hepatitis.

Clinical Parameters		Alcoholic Hepatitis (*n* = 210)
Sex (% male), *n* (%), *n* = 208		138 (66.3%)
Age (years), *n* = 198		49.3 (26.4–74.8)
BMI (kg/m^2^), *n* = 165		28.2 (16.2–48.3)
Laboratory parameter		
Creatinine (mg/dL), *n* = 195		1.1 (0.3–8.1)
Bilirubin (mg/dL), *n* = 194		16.0 (2.5–51.8)
AST (IU/L), *n* = 195		165.2 (34.0–1858.0)
ALT (IU/L), *n* = 194		57.5 (14.0–404.0)
Albumin (g/dL), *n* = 188		2.6 (1.1–4.2)
INR, *n* = 194		1.9 (0.8–7.6)
GGT (IU/L), *n* = 114		602.6 (33.0–3650.0)
Platelet count (10^9^/L), *n* = 191		136.5 (12.2–447.0)
Alkaline phosphatase (U/L), *n* = 192		207.6 (21.2–1153.0)
Prothrombin time, s, *n* = 168		29.3 (9.0–141.0)
Sodium (mEq/L), *n* = 194		133.0 (106.0–148.0)
FIB-4, *n* = 189		11.5 (0.7–116.0)
FIB-4 > 3.25 (F3-F4), *n* (%)		171 (90.5%)
Treatment at admission		
Steroids, *n* (%), *n* = 193		83 (43.0%)
Antibiotics, *n* (%), *n* = 193		53 (27.5%)
Proton pump inhibitors, *n* (%), *n* = 98		13 (13.3%)
Infections, *n* (%), *n* = 158		41 (25.9%)
Clinical scores and outcome		
MELD, median (range), *n* = 192		26.8 (10.1–48.6)
MELD > 21, *n* (%)		158 (82.3%)
30-day mortality (*n* = 210)		31 (14.8%)
90-day mortality (*n* = 158)		54 (34.2%)
Histology		
Liver biopsy available, *n* (%), *n* = 197		120 (60.9%)
Stage of fibrosis, *n* (%), *n* = 118	0	3 (2.5%)
	1	3 (2.5%)
	2	15 (12.7%)
	3	18 (15.3%)
	4	79 (66.9%)

Note: Values presented are median with range in parentheses for continuous variables or number and percentage in parentheses for categorical variables. Percentages were calculated based on the actual number of patients in each group, when data were available. The number of subjects for which data were available is indicated in the first column. MELD, model for end-stage liver disease; BMI, body mass index; AST, aspartate aminotransferase; ALT, alanine aminotransferase; INR, international normalized ratio; GGT, gamma-glutamyl transferase; FIB-4, fibrosis-4 index. Fibrosis stage, 0 no fibrosis, 1 portal fibrosis, 2 expansive periportal fibrosis, 3 bridging fibrosis, 4 cirrhosis.

**Table 2:** Variables with the top 11 average feature importance in each dataset.

	**Clinical Data**	**Clinical Data + Bacteria + MetaCyc Pathways**	**Clinical Data + Fungi**
30-day	age	international normalized ratio	creatinine
	creatinine	creatinine	international normalized ratio
	bilirubin	sodium	bilirubin
	albumin	PWY-6125: superpathway of guanosine nucleotides de novo biosynthesis II	sodium
	international normalized ratio	DTDPRHAMSYN-PWY: dTDP-L-rhamnose biosynthesis I	Aspergillus
	alanine transaminase	PWY-7229: superpathway of adenosine nucleotides de novo biosynthesis I	alkaline phosphatase
	alkaline phosphatase	PWY-7222: guanosine deoxyribonucleotides de novo biosynthesis II	age
	platelet count	PWY-7228: superpathway of guanosine nucleotides de novo biosynthesis I	aspartate transaminase
	white blood cell count	PANTO-PWY: phosphopantothenate biosynthesis I	platelets
	aspartate transaminase	PWY-6126: superpathway of adenosine nucleotides de novo biosynthesis II	alanine transaminase
	sodium	PWY-7220: adenosine deoxyribonucleotides de novo biosynthesis II	white blood cell count
90-day	age	international normalized ratio	creatinine
	creatinine	creatinine	bilirubin
	bilirubin	bilirubin	sodium
	albumin	sodium	international normalized ratio
	international normalized ratio	PWY-7229: superpathway of adenosine nucleotides de novo biosynthesis I	age
	alanine transaminase	DTDPRHAMSYN-PWY: dTDP-L-rhamnose biosynthesis I	albumin
	alkaline phosphatase	GLUCOSE1PMETAB-PWY: glucose and glucose-1 phosphate degradation	alkaline phosphatase
	platelet count	PWY-5989: stearate biosynthesis II bacteria and plants	aspartate transaminase
	white blood cell count	Clostridium nexile	platelets
	aspartate transaminase	PWY0-1297: superpathway of purine deoxyribonucleosides degradation	white blood cell count
	sodium	PWY-6125: superpathway of guanosine nucleotides de novo biosynthesis II	alanine transaminase
	**Clinical Data + Virus**	**Clinical Data + ELISA**	**Clinical Data + Metabolites + Lipids**
30-day	creatinine	creatinine	creatinine
	international normalized ratio	international normalized ratio	L-Hydroxyarginine
	bilirubin	bilirubin	bilirubin
	sodium	Zonulin	Urea
	Epsilon15 virus	albumin	Pseudo uridine
	aspartate transaminase	sodium	Maltose
	P22 virus	Lipopolysaccharide binding protein	Erythritol
	Lambda virus	Anti-Saccharomyces cerevisiae antibodies	Metabolite creatinine
	alkaline phosphatase	age	S-adenosyl homocysteine
	alanine transaminase	platelets	L-Carnitine
	age	alanine transaminase	Adenine
90-day	creatinine	creatinine	creatinine
	international normalized ratio	international normalized ratio	international normalized ratio
	bilirubin	bilirubin	L-Homocitrulline
	sodium	age	Lyxitol
	age	Anti-Saccharomyces cerevisiae antibodies	Pseudo uridine
	albumin	sodium	L-Hydroxyarginine
	alanine transaminase	white blood cell count	Adipoyl-L-carnitine
	alkaline phosphatase	Zonulin	Asymmetric dimethylarginine
	Epsilon15 virus	albumin	sodium
	platelets	Lipopolysaccharide binding protein	Acyl carnitines
	white blood cell count	alkaline phosphatase	Kynurenic acid

**Table 3:** Summary of AUC scores for each dataset.

	Model	Clinical Data	Clinical Data + Bacteria + Metacyc Pathways	Clinical Data + Fungi	Clinical Data + Virus	Clinical Data + Metabolites + Lipids	Clinical Data + ELISA
30-day Mortality	LR(MELD)	0.78	0.79	0.72	0.72	0.77	0.77
	LR	0.74	0.59	0.56	***0.87***	0.65	0.72
	SVM	0.76	0.68	**0.86**	**0.80**	0.74	0.74
	RF	**0.79**	**0.83**	0.45	**0.74**	0.73	0.76
	GB	**0.81**	***0.87***	0.35	0.69	0.71	0.69
90-day Mortality	LR(MELD)	0.82	0.63	0.25	0.67	0.83	0.79
	LR	0.80	**0.67**	**0.55**	0.62	0.67	0.64
	SVM	0.80	**0.69**	**0.73**	0.63	0.69	0.64
	RF	0.80	0.54	**0.68**	0.53	0.78	0.71
	GB	0.79	0.47	***0.87***	0.58	0.71	0.71

Note: LR: logistic regression, SVM: support vector machine, RF: random forest, GB: gradient boosting, LR (MELD): logistic regression model using MELD score only based on the same subset of patients as each dataset. Clinical data day-30 deceased group *n* = 31, alive group *n* = 179. Day-90 deceased group *n* = 54, alive group *n* = 104. Clinical data, bacteria and MetaCyc pathways day 30: deceased group *n* = 8; alive group *n* = 65. Day 90: deceased group *n* = 13; alive group *n* = 40. Clinical data and fungi day 30: deceased group *n* = 5; alive group *n* = 49. Day 90: deceased group *n* = 9; alive group *n* = 30. Clinical data and virus day 30: deceased group *n* = 8; alive group *n* = 68. Day 90: deceased group *n* = 14; alive group *n* = 42. Clinical data, metabolites and lipids day 30: deceased group *n* = 19; alive group *n* = 99. Day 90: deceased group *n* = 33; alive group *n* = 57. Clinical data and serum biomarkers day 30: decreased group *n* = 20; alive group *n* = 118. Day 90: deceased group *n* = 36; alive group *n* = 68. Bold: ROC score higher than LR (MELD) model in each dataset. *Italic*: highest AUC for 30-day or 90-day mortality.

## Data Availability

Code and machine learning models are available on Github (https://github.com/morris16206/Alcoholic-hepatitis (accessed on 12 August 2021)). Raw 16S rRNA sequencing reads can be found in the National Center for Biotechnology Information (NCBI) SRA associated with Bioproject PRJNA525701. Fungal sequencing data can be found under BioProject PRJNA517994. Shotgun Metagenomics sequence data were deposited in the European Nucleotide Archive under accession numbers ERP106878. Metabolomics and lipidomics datasets are provided in this manuscript as supplementary files.

## Supplementary Materials

The following are available online at https://www.mdpi.com/article/10.3390/metabo12010041/s1, Figure S1: A flow chart for machine learning methodology; Table S1: Metabolomics dataset; Table S2: Lipidomics dataset.

## References

[1] Osna N.A., Donohue T.M., Kharbanda K.K. (2017). Alcoholic Liver Disease: Pathogenesis and Current Management. Alcohol. Res..

[2] Sandahl T.D., Jepsen P., Thomsen K.L., Vilstrup H. (2011). Incidence and Mortality of Alcoholic Hepatitis in Denmark 1999–2008: A Nationwide Population Based Cohort Study. J. Hepatol..

[3] Dominguez M., Rincón D., Abraldes J.G., Miquel R., Colmenero J., Bellot P., García-Pagán J.-C., Fernández R., Moreno M., Bañares R. (2008). A New Scoring System for Prognostic Stratification of Patients with Alcoholic Hepatitis. Am. J. Gastroenterol..

[4] Llopis M., Cassard A.M., Wrzosek L., Boschat L., Bruneau A., Ferrere G., Puchois V., Martin J.C., Lepage P., Le Roy T. (2016). Intestinal Microbiota Contributes to Individual Susceptibility to Alcoholic Liver Disease. Gut.

[5] Philips C.A., Phadke N., Ganesan K., Ranade S., Augustine P. (2018). Corticosteroids, Nutrition, Pentoxifylline, or Fecal Microbiota Transplantation for Severe Alcoholic Hepatitis. Indian J. Gastroenterol..

[6] Duan Y., Llorente C., Lang S., Brandl K., Chu H., Jiang L., White R.C., Clarke T.H., Nguyen K., Torralba M. (2019). Bacteriophage Targeting of Gut Bacterium Attenuates Alcoholic Liver Disease. Nature.

[7] Chu H., Duan Y., Lang S., Jiang L., Wang Y., Llorente C., Liu J., Mogavero S., Bosques-Padilla F., Abraldes J.G. (2020). The Candida Albicans Exotoxin Candidalysin Promotes Alcohol-Associated Liver Disease. J. Hepatol..

[8] Lang S., Duan Y., Liu J., Torralba M.G., Kuelbs C., Ventura-Cots M., Abraldes J.G., Bosques-Padilla F., Verna E.C., Brown R.S. (2020). Intestinal Fungal Dysbiosis and Systemic Immune Response to Fungi in Patients With Alcoholic Hepatitis. Hepatology.

[9] Jang L., Lang S., Duan Y., Zhang X., Gao B., Chopyk J., Schwanemann L.K., Ventura-Cots M., Bataller R., Bosques-Padilla F. (2020). Intestinal Virome in Patients With Alcoholic Hepatitis. Hepatology.

[10] Saha B., Tornai D., Kodys K., Adejumo A., Lowe P., McClain C., Mitchell M., McCullough A., Dasarathy S., Kroll-Desrosiers A. (2019). Biomarkers of Macrophage Activation and Immune Danger Signals Predict Clinical Outcomes in Alcoholic Hepatitis. Hepatology.

[11] Heelan B.T., Allan S., Barnes R.M. (1991). Identification of a 200-KDa Glycoprotein Antigen of Saccharomyces Cerevisiae. Immunol. Lett..

[12] Wang L., Llorente C., Hartmann P., Yang A.-M., Chen P., Schnabl B. (2015). Methods to Determine Intestinal Permeability and Bacterial Translocation during Liver Disease. J. Immunol. Methods.

[13] Fasano A. (2011). Zonulin and Its Regulation of Intestinal Barrier Function: The Biological Door to Inflammation, Autoimmunity, and Cancer. Physiol. Rev..

[14] Gutsmann T., Müller M., Carroll S.F., MacKenzie R.C., Wiese A., Seydel U. (2001). Dual Role of Lipopolysaccharide (LPS)-Binding Protein in Neutralization of LPS and Enhancement of LPS-Induced Activation of Mononuclear Cells. Infect. Immun..

[15] Chiappini F., Coilly A., Kadar H., Gual P., Tran A., Desterke C., Samuel D., Duclos-Vallée J.-C., Touboul D., Bertrand-Michel J. (2017). Metabolism Dysregulation Induces a Specific Lipid Signature of Nonalcoholic Steatohepatitis in Patients. Sci. Rep..

[16] Caussy C., Tripathi A., Humphrey G., Bassirian S., Singh S., Faulkner C., Bettencourt R., Rizo E., Richards L., Xu Z.Z. (2019). A Gut Microbiome Signature for Cirrhosis Due to Nonalcoholic Fatty Liver Disease. Nat. Commun..

[17] (2012). European Association for the Study of Liver EASL Clinical Practical Guidelines: Management of Alcoholic Liver Disease. J. Hepatol..

[18] Forrest E.H., Atkinson S.R., Richardson P., Masson S., Ryder S., Thursz M.R., Allison M. (2018). STOPAH trial Management Group Application of Prognostic Scores in the STOPAH Trial: Discriminant Function Is No Longer the Optimal Scoring System in Alcoholic Hepatitis. J. Hepatol..

[19] Wu C.-C., Yeh W.-C., Hsu W.-D., Islam M.M., Nguyen P.A.A., Poly T.N., Wang Y.-C., Yang H.-C., Jack Li Y.-C. (2019). Prediction of Fatty Liver Disease Using Machine Learning Algorithms. Comput. Methods Programs Biomed..

[20] Wei R., Wang J., Wang X., Xie G., Wang Y., Zhang H., Peng C.-Y., Rajani C., Kwee S., Liu P. (2018). Clinical Prediction of HBV and HCV Related Hepatic Fibrosis Using Machine Learning. EBioMedicine.

[21] Oh T.G., Kim S.M., Caussy C., Fu T., Guo J., Bassirian S., Singh S., Madamba E.V., Bettencourt R., Richards L. (2020). A Universal Gut Microbiome-Derived Signature Predicts Cirrhosis. Cell Metab..

[22] Samant S., Lee H., Ghassemi M., Chen J., Cook J.L., Mankin A.S., Neyfakh A.A. (2008). Nucleotide Biosynthesis Is Critical for Growth of Bacteria in Human Blood. PLoS Pathog..

[23] Gao B., Lang S., Duan Y., Wang Y., Shawcross D.L., Louvet A., Mathurin P., Ho S.B., Stärkel P., Schnabl B. (2019). Serum and Fecal Oxylipins in Patients with Alcohol-Related Liver Disease. Dig. Dis. Sci..

[24] Gao B., Emami A., Zhou R., Lang S., Duan Y., Wang Y., Jiang L., Loomba R., Brenner D., Stärkel P. (2020). Functional Microbial Responses to Alcohol Abstinence in Patients with Alcohol Use Disorder. Front. Physiol..

[25] Truong D.T., Franzosa E.A., Tickle T.L., Scholz M., Weingart G., Pasolli E., Tett A., Huttenhower C., Segata N. (2015). MetaPhlAn2 for Enhanced Metagenomic Taxonomic Profiling. Nat. Methods.

[26] Franzosa E.A., McIver L.J., Rahnavard G., Thompson L.R., Schirmer M., Weingart G., Lipson K.S., Knight R., Caporaso J.G., Segata N. (2018). Species-Level Functional Profiling of Metagenomes and Metatranscriptomes. Nat. Methods.

[27] Caspi R., Billington R., Keseler I.M., Kothari A., Krummenacker M., Midford P.E., Ong W.K., Paley S., Subhraveti P., Karp P.D. (2020). The MetaCyc Database of Metabolic Pathways and Enzymes-a 2019 Update. Nucleic Acids Res..

[28] Conceição-Neto N., Yinda K.C., Van Ranst M., Matthijnssens J. (2018). NetoVIR: Modular Approach to Customize Sample Preparation Procedures for Viral Metagenomics. Methods Mol. Biol..

[29] Kostic A.D., Ojesina A.I., Pedamallu C.S., Jung J., Verhaak R.G.W., Getz G., Meyerson M. (2011). PathSeq: Software to Identify or Discover Microbes by Deep Sequencing of Human Tissue. Nat. Biotechnol..

[30] Gao B., Lue H.-W., Podolak J., Fan S., Zhang Y., Serawat A., Alumkal J.J., Fiehn O., Thomas G.V. (2019). Multi-Omics Analyses Detail Metabolic Reprogramming in Lipids, Carnitines, and Use of Glycolytic Intermediates between Prostate Small Cell Neuroendocrine Carcinoma and Prostate Adenocarcinoma. Metabolites.

[31] Skogerson K., Wohlgemuth G., Barupal D.K., Fiehn O. (2011). The Volatile Compound BinBase Mass Spectral Database. BMC Bioinform..

[32] Tsugawa H., Cajka T., Kind T., Ma Y., Higgins B., Ikeda K., Kanazawa M., VanderGheynst J., Fiehn O., Arita M. (2015). MS-DIAL: Data-Independent MS/MS Deconvolution for Comprehensive Metabolome Analysis. Nat. Methods.

[33] Schrimpe-Rutledge A.C., Codreanu S.G., Sherrod S.D., McLean J.A. (2016). Untargeted Metabolomics Strategies—Challenges and Emerging Directions. J. Am. Soc. Mass Spectrom..

[34] DeFelice B.C., Mehta S.S., Samra S., Čajka T., Wancewicz B., Fahrmann J.F., Fiehn O. (2017). Mass Spectral Feature List Optimizer (MS-FLO): A Tool To Minimize False Positive Peak Reports in Untargeted Liquid Chromatography–Mass Spectroscopy (LC-MS) Data Processing. Anal. Chem..

[35] Hosmer D.W., Lemeshow S., Sturdivant R.X. (2013). Applied Logistic Regression.

[36] Cortes C., Vapnik V. (1995). Support-Vector Networks. Mach. Learn..

[37] Breiman L. (2001). Random Forests. Mach. Learn..

[38] Friedman J.H. (2001). Greedy Function Approximation: A Gradient Boosting Machine. Ann. Statist..

[39] Azur M.J., Stuart E.A., Frangakis C., Leaf P.J. (2011). Multiple Imputation by Chained Equations: What Is It and How Does It Work?. Int. J. Methods Psychiatr. Res..

[40] Chawla N.V., Bowyer K.W., Hall L.O., Kegelmeyer W.P. (2002). SMOTE: Synthetic Minority over-Sampling Technique. J. Artif. Intell. Res..

[41] Wright M.N., Ziegler A. (2017). Ranger: A Fast Implementation of Random Forests for High Dimensional Data in C++ and R. J. Stat. Softw..

